# Metabolomics in melon: A new opportunity for aroma analysis

**DOI:** 10.1016/j.phytochem.2013.12.010

**Published:** 2014-03

**Authors:** J. William Allwood, William Cheung, Yun Xu, Roland Mumm, Ric C.H. De Vos, Catherine Deborde, Benoit Biais, Mickael Maucourt, Yosef Berger, Arthur A. Schaffer, Dominique Rolin, Annick Moing, Robert D. Hall, Royston Goodacre

**Affiliations:** a School of Chemistry, University of Manchester, 131 Princess Street, Manchester M1 7DN, UK; b Manchester Centre for Integrative Systems Biology, Manchester Institute of Biotechnology, University of Manchester, 131 Princess Street, Manchester M1 7DN, UK; c School of Biosciences, University of Birmingham, Edgbaston, Birmingham B15 2TT, UK; d Plant Research International, P.O. Box 16, 6700 AA Wageningen, Netherlands; e Netherlands Metabolomics Centre, Einsteinweg 55, 2333 CC Leiden, Netherlands; f Centre for BioSystems Genomics, P.O. Box 98, 6700AB Wageningen, Netherlands; g INRA, UMR1332 Biologie du Fruit et Pathologie, INRA – Université de Bordeaux, Centre INRA de Bordeaux, IBVM, CS20032, F-33140 Villenave d’Ornon, France; h Metabolome Facility of Bordeaux Functional Genomics Centre, Centre INRA de Bordeaux, IBVM, F-33140 Villenave d’Ornon, France; i Université de Bordeaux, UMR1332 Biologie du Fruit et Pathologie, INRA – Université de Bordeaux, Centre INRA de Bordeaux, IBVM, CS20032, F-33140 Villenave d’Ornon, France; j Agricultural Research Organisation (ARO), The Volcani Center, Bet Dagan 50250, Israel

**Keywords:** *Cucumis melo*, Volatile organic compound (VOC), Aroma, Amino acid, Thermal desorption (TD), Solid phase micro extraction (SPME), Gas chromatography mass spectrometry (GC–MS), Proton-nuclear magnetic resonance spectroscopy (^1^H NMR), Metabolomics

## Abstract

*Cucumis melo* fruit is highly valued for its sweet and refreshing flesh, however the flavour and value are also highly influenced by aroma as dictated by volatile organic compounds (VOCs). A simple and robust method of sampling VOCs on polydimethylsiloxane (PDMS) has been developed. Contrasting cultivars of *C. melo* subspecies *melo* were investigated at commercial maturity: three cultivars of var. Cantalupensis group Charentais (cv. Cézanne, Escrito, and Dalton) known to exhibit differences in ripening behaviour and shelf-life, as well as one cultivar of var. Cantalupensis group Ha’Ogan (cv. Noy Yisre’el) and one non-climacteric cultivar of var. Inodorus (cv. Tam Dew). The melon cultivar selection was based upon fruits exhibiting clear differences (cv. Noy Yisre’el and Tam Dew) and similarities (cv. Cézanne, Escrito, and Dalton) in flavour. In total, 58 VOCs were detected by thermal desorption (TD)-GC–MS which permitted the discrimination of each cultivar via Principal component analysis (PCA). PCA indicated a reduction in VOCs in the non-climacteric cv. Tam Dew compared to the four Cantalupensis cultivars. Within the group Charentais melons, the differences between the short, mid and long shelf-life cultivars were considerable. ^1^H NMR analysis led to the quantification of 12 core amino acids, their levels were 3–10-fold greater in the Charentais melons, although they were reduced in the highly fragrant cv. Cézanne, indicating their role as VOC precursors. This study along with comparisons to more traditional labour intensive solid phase micro-extraction (SPME) GC–MS VOC profiling data has indicated that the high-throughput PDMS method is of great potential for the assessment of melon aroma and quality.

## Highlights

• The aroma of *Cucumis melo* fruit highly influences financial value.
• A robust method of fruit aroma sampling followed by TD-GC–MS detection was developed.
• Aroma was reduced in non-climacteric fruit, in climacteric melons shelf-life greatly affected aroma.
• Comparisons to SPME-GC–MS has indicated the validity of the new method.
• The new method is suited for rapid assessment of fruit aroma and quality.

## Graphical abstract

Melon fruit value is highly influenced by aroma. A simple and robust method of trapping volatile organic compounds combined with detection via thermal desorption (TD)-GC–MS has been developed. Principal component analysis of aroma profiles permitted the characterisation of distinct and closely related melon cultivars according to aromatic quality and shelf life.

**Figure:**
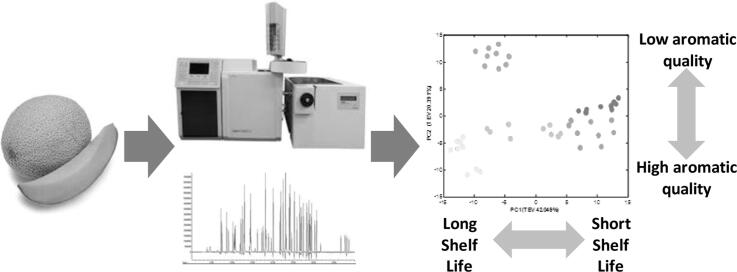

## Introduction

The melon (*Cucumis melo* L.) belongs to the Cucurbitaceae family, which contains numerous species differing greatly in fruit size (several grams to kilograms), shape (round to elongated) and organoleptic properties (bitter to sweet) (Stepansky et al., 1999a). Climacteric *C. melo* cultivars (Hadfiel et al., 1995) such as the var. *cantalupensis* (groups Ha’Ogan and Charentais) are highly prized for their sweet, refreshing, and aromatic flesh, whereas non-climacteric *C. melo* such as the var. *inodorus* melons lack aroma (Stepansky et al., 1999a, Stepansky et al., 1999b). The aroma of melon fruit is dictated by the content of volatile organic compounds (VOCs) (Aubert and Bourger, 2004, Berger, 1991, Buttery et al., 1982, Fallik et al., 2001, Kourkoutas et al., 2006, Yabumoto et al., 1977). From a chemical perspective VOCs represent a heterogeneous group of compounds, with aromatic, hetero-aromatic, branched- and straight-chain backbones, with diverse chemical groups as for example hydroxyl, carboxyl, carbonyl, amine, ester, lactone, and thiol functions (Schwab et al., 2008). The detection of VOCs has classically been achieved by means of Gas chromatography–mass spectrometry (GC–MS) (Dewulf et al., 2002) since GC lends itself to the separation of sample components based upon volatility and thus no form of chemical derivatisation is required. VOC samples are introduced to the GC–MS via a number of methods including both solid phase micro-extraction (SPME) (Beaulieu and Grimm, 2001, Song et al., 1997) and direct thermal desorption (TD) (Riazanskaia et al., 2008, Xu et al., 2010).

Contrasting test materials for this investigation, which was performed as part of the EU Frame Work VI META-PHOR project (http://www.meta-phor.eu/) (Allwood et al., 2009, Biais et al., 2009, Hall et al., 2008, Moing et al., 2011), were provided by the Israel Agricultural Research Organisation (ARO), two green fleshed melon cultivars, *C. melo* var. *cantalupensis* group Ha’Ogan cv. Noy Yisre’el (henceforth called Noy Yisre’el), and a non-aromatic fruit, *C. melo* var. *inodorus* cv. Tam Dew (henceforth called Tam Dew) (Stepansky et al., 1999a), and by France CEFEL who provided three orange fleshed *C. melo* var. *cantalupensis* group Charentais melon cultivars known as (and henceforth called) Cézanne, Escrito, and Dalton, which exhibit differences in ripening behaviour and shelf-life (Aubert and Bourger, 2004). Cézanne is the most aromatic cultivar but has a very short shelf-life, Escrito has a mid shelf-life and is less aromatic, Dalton has the longest shelf-life and is considered to be the least aromatic (Aubert and Bourger, 2004, Dumoulin and Odet, 1998). Previous GC–TOF/MS and ^1^H NMR spatial analysis of extracted polar metabolites in Cézanne and Escrito melon fruit indicated that the inner mesocarp was hypoxic and produced ethanol, it also contained high concentrations of specific sugars and amino acids (Biais et al., 2009, Biais et al., 2010), which are known to be key precursors for the VOCs responsible for fruit aroma (Gonda et al., 2013, Schwab et al., 2008).

Melon VOCs have been extensively investigated in many varieties, especially with regard to how aroma profiles alter during fruit ripening (Beaulieu and Grimm, 2001, Buttery et al., 1982, Fallik et al., 2001, Homatidou et al., 1992, Kourkoutas et al., 2006, Schieberle et al., 1990, Vallone et al., 2013, Wang et al., 1996, Yabumoto et al., 1977). Other investigations have focused upon the aroma profiles of antisense-ACC oxidase expressing plants that revealed massive reductions in VOCs (Ayub et al., 1996, Bauchot et al., 1998, Flores et al., 2002). Although aroma profiles have been previously investigated in Charentais melon varieties (Homatidou et al., 1992, Bauchot et al., 1998, Flores et al., 2002), only one previous study has focused upon the aroma profiles of Charentais with respect to shelf-life (Aubert and Bourger, 2004). The aim of this study was firstly to develop a simple and inexpensive screening method of sampling melon VOCs that could also be appropriate for application to other fruits, and secondly to validate this method by investigating the aroma variability of a series of diverse melon cultivars. Subsequently, ^1^H NMR was applied for the quantification of amino acids, since they are known to act as precursors for many volatile constituents that contribute to aroma (Gonda et al., 2013, Schwab et al., 2008). While the fruits used are equivalent to supermarket availability, they were grown under different conditions between the countries of origin. Consequently, the analysis of the melon fruits was considered first as a whole and second as two independent comparisons. The first comparison focused on the two diverse Israeli cultivars and posed the question whether PDMS derived data are able to discriminate typical climacteric and non-climacteric varieties. The second comparison focused on the closely related French Charentais melons since they posed more of an analytical challenge to discriminate and also posed the question as to which VOC components differed between the short, mid and long shelf-life fruit.

## Results & discussion

### A reliable and robust simplified method of passively sampling melon VOCs

**Fig. 1:**
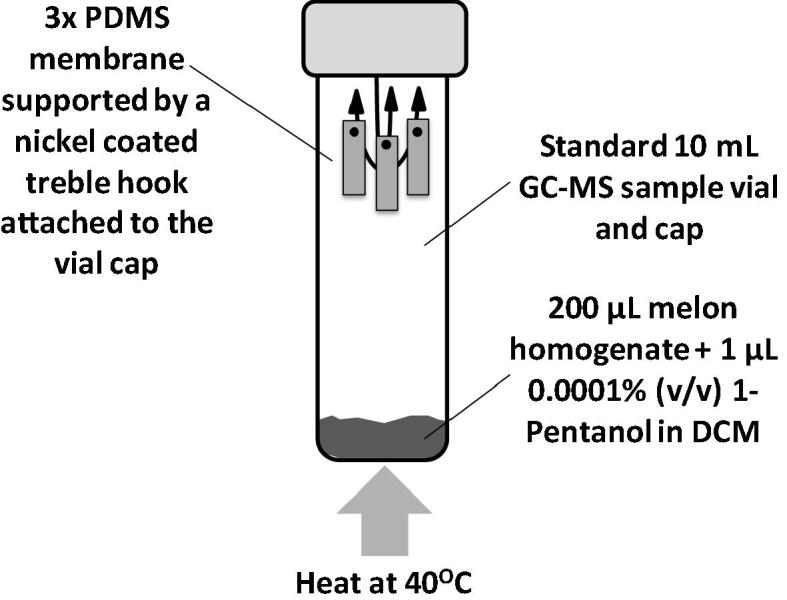
Sampling of melon volatile organic compounds using PDMS membranes. An illustration as to how sampling melon VOCs upon three technical replicate PDMS membranes is achieved.

**Fig. 2:**
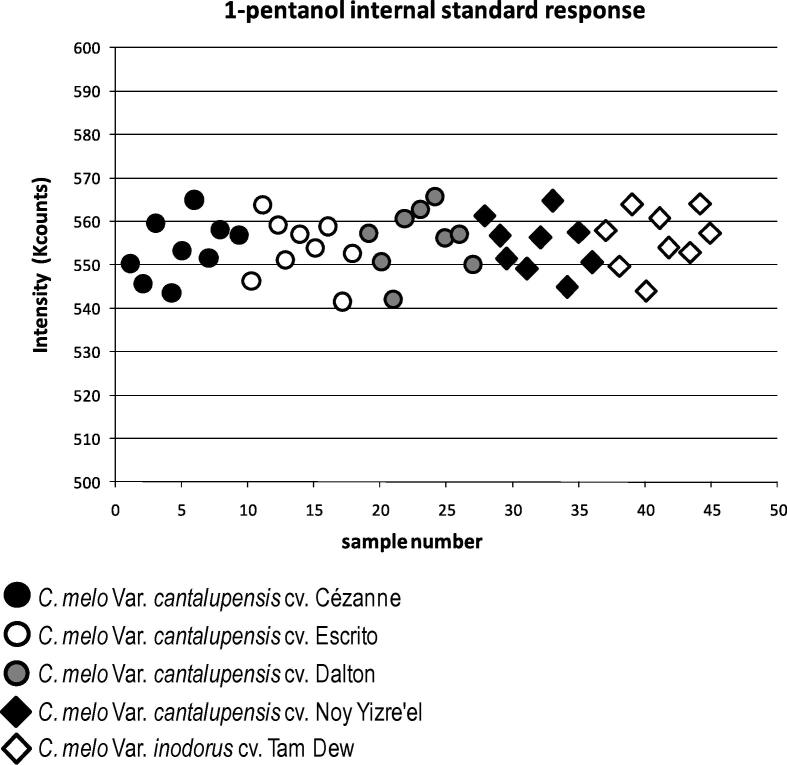
Reproducibility of PDMS patch volatile organic compound sampling and TD-GC–MS analysis. The 1-Pentanol internal standard showed a highly reproducible response (based upon the peak area in single ion monitoring mode) indicating excellent robustness in terms of both the PDMS patch sampling method (technical reproducibility) and the TD-GC–MS systems analytical reproducibility. Thus, high confidence was given with respect to the simple sampling method that PDMS patch ambient absorption of VOCs provides, so much so that normalisation of each sample to their respective internal standard response was not deemed necessary.

The first aim of the study was to develop a simple and reproducible screening method for the ambient sampling of melon VOCs, that could be applied to the future analyses of any potential fruit or plant derived foodstuff. PDMS membrane was selected, since the sample collection routine simply involved the gentle warming of a set volume of melon homogenate within a sealed vial to liberate VOCs that were subsequently trapped upon three technical replicate PDMS membranes suspended from the vial lid (Fig. 1). Fears were raised that such a simplified method of VOC trapping would not be reproducible, therefore a set volume of 0.0001% 1-pentanol was added to the melon homogenate to serve as an internal standard. Based upon the peak area in single ion monitoring mode, the 1-pentanol signal was stable throughout the experiment for all blanks, technical and biological replicate samples regardless of the melon cultivar (standard deviation = 6.76; standard error = 1.01; Fig. 2). Therefore confidence was installed that the passive melon VOC sampling technique was not only easy to perform but was also highly reproducible.

### PDMS is a suitable sorbent material for trapping a wide range of melon VOCs

Analysis of the PDMS trapped VOCs was performed by means of TD-GC–MS. In total 58 compounds were detected, 47 of which were putatively identified with high confidence (Table 1). These compounds fell into many classes, most significantly perhaps were the esters (*n *= 19), alcohols (*n *= 6), sulfur compounds (*n *= 3), and aldehydes (*n *= 2), although several other classes were detected including ketones, alkanes, akenes and alkynes. A greater number of aromatic compounds were detected when we applied a standard SPME approach (Verhoeven et al., 2011) to an identical sample set (Supplementary Fig. 1). However, the larger number of detected VOCs compared to the PDMS approach most likely relates to differences in the extraction protocol (15 × larger sample volumes and the use of inorganic salts) rather than trapping efficiency or detection sensitivity. The supplementary SPME data also confirm the presence of many of the identified VOCs detected using the PDMS approach, boding well for its application as a rapid screening approach. Comparisons of our PDMS and SPME data for the same compounds also reveals some differences in varietal distribution which again can be attributed to relative differences in trapping efficiencies by the different adsorbents (Supplementary Figs. 1 and 2). On the basis of the major VOC groups (alcohols, esters, sulfur compounds and aldehydes), 22 out of a total of 30 compounds detected via the PDMS method were also detected in the total of 75 SPME compounds of the same respective chemical classes, indicating the complementary nature of the two methods as well as the greater sensitivity of SPME.

**Table 1:** Volatile organic compounds (VOCs) detected via PDMS TD-GC–MS of melon fruit samples.

PCA reference No.	VOC ID	Compound class	PubChem identifier	PCA significant	UVA (Kruskal–Wallis) *P* value	Cézanne average	Cézanne SE	Escrito Average	Escrito SE	Dalton average	Dalton SE	Noy Yizreel average	Noy Yizreel SE	Tam Dew average	Tam Dew SE
10	2-Methyl-1-butanol	Alcohol	CID: 8723	Y	1.17E-07	14.07	0.079	13.78	0.023	12.07	0.159	14.11	0.073	10.85	0.063
23	(Z)-3-hexen-1-ol	Alcohol	CID: 10993	Y	5.88E-08	11.90	0.076	9.78	0.064	9.39	0.109	12.00	0.071	10.71	0.064
28	1-Hexanol	Alcohol	CID: 8103	Y	3.23E-08	13.16	0.035	12.68	0.031	11.25	0.099	13.41	0.061	10.72	0.048
35	2-(4-Methoxyphenyl)propan-2-ol	Alcohol	CID: 81930	Y	1.99E-01	7.86	0.404	5.20	1.335	8.10	0.220	8.30	1.149	6.63	1.703
46	1-Octanol	Alcohol	CID: 957	Y	6.78E-06	10.03	0.052	9.83	0.078	9.69	0.094	12.70	0.075	9.69	0.058
52	Trans-2-cis-6-Nonadien-1-ol	Alcohol	CID: 5320191	Y	3.10E-06	13.03	0.078	11.93	0.028	11.98	0.104	12.14	0.092	12.27	0.034
		TOTAL ALCOHOLS		NA	2.88E-06	70.06	0.396	63.20	1.379	62.49	0.520	72.65	1.192	60.87	1.691

7	Propyl acetate	Ester	CID: 7997	Y	4.09E-08	14.50	0.087	14.00	0.055	12.60	0.191	13.13	0.139	10.40	0.113
11	Ethyl propanoate	Ester	CID: 7749	Y	3.67E-08	13.07	0.092	12.11	0.063	10.80	0.244	10.47	0.212	7.00	0.223
15	2-Methyl-methyl butanoate	Ester	CID: 13357	Y	5.26E-04	12.82	0.231	11.55	0.066	12.20	0.158	11.51	0.234	11.69	0.162
16	2,2-Dimethylbutanoate	Ester	CID: 6951315	Y	1.77E-07	12.59	0.153	11.02	0.062	10.75	0.202	10.30	0.088	8.43	0.270
18	Propyl propanoate	Ester	CID: 7803	Y	5.39E-04	13.82	0.134	14.01	0.028	13.89	0.080	13.91	0.116	12.56	0.148
19	Butyl acetate	Ester	CID: 31272	Y	5.58E-08	14.30	0.104	13.71	0.043	12.86	0.135	14.40	0.070	11.49	0.150
20	Ethyl butanoate	Ester	CID: 7762	Y	3.19E-08	14.38	0.150	12.67	0.053	11.29	0.234	11.01	0.085	9.13	0.068
22	2-Methyl-ethyl butanoate	Ester	CID: 24020	Y	3.61E-08	13.82	0.163	12.38	0.057	10.68	0.249	11.04	0.064	6.86	0.198
24	Diethyl 2-hydroxy-3-methylbutanedioate	Ester	CID: 582908	Y	1.11E-07	10.42	0.615	6.87	0.053	6.62	0.116	5.37	0.109	5.45	0.128
29	2-Methylbutyl acetate	Ester	CID: 12209	Y	4.94E-06	13.22	0.127	12.12	0.040	12.87	0.076	13.00	0.061	12.47	0.123
33	2-Methylpropyl butanoate	Ester	CID: 10885	Y	1.23E-06	8.11	0.228	6.92	0.407	7.57	0.117	11.34	0.089	4.42	1.112
39	Ethyl hexanoate	Ester	CID: 31265	Y	1.65E-06	0.00	0.000	1.47	0.122	1.23	0.353	2.16	0.437	7.01	0.061
41	(Z)-3-Hexenyl acetate	Ester	CID: 5363388	Y	2.07E-08	12.22	0.128	9.69	0.031	10.68	0.128	13.67	0.065	11.32	0.098
42	2-Methylpentyl acetate	Ester	CID: 24625	Y	8.10E-07	12.57	0.194	11.59	0.036	11.84	0.180	14.44	0.063	11.47	0.115
45	Eucalyptol	Ester/monoterpenoid	CID: 2758	Y	1.43E-07	10.75	0.064	10.43	0.024	10.07	0.062	10.65	0.064	11.37	0.118
50	2,4-Diacetoxypentane	Ester	CID: 139007	Y	3.23E-08	11.39	0.091	9.40	0.052	10.19	0.126	10.58	0.097	12.99	0.095
51	Phenylmethyl acetate	Ester	CID: 8785	Y	4.77E-07	6.61	0.261	5.07	0.729	7.68	0.602	10.11	0.172	13.62	0.142
55	Phenylmethyl acetate	Ester	CID: 8785	Y	4.64E-08	10.93	0.146	9.58	0.020	11.66	0.288	12.84	0.070	13.81	0.036
58	(Z)-3-Octenyl acetate	Ester	CID: 5363205	Y	2.77E-06	6.99	0.111	3.00	0.958	5.00	0.277	10.89	0.085	5.37	0.831
		TOTAL ESTERS		NA	1.09E-06	212.51	2.096	187.59	1.413	190.47	2.100	210.82	1.407	186.87	1.357

37	Benzaldehyde	Aldehyde	CID: 240	Y	5.82E-08	9.91	0.056	9.41	0.042	11.87	0.223	8.89	0.089	9.85	0.087
48	2-Nonenal, (E)-	Aldehyde	CID: 5283335	N	4.98E-07	10.61	0.106	9.74	0.072	9.98	0.112	11.77	0.072	10.19	0.084
		TOTAL ALDEHYDES		NA	4.30E-07	20.51	0.127	19.16	0.090	21.85	0.241	20.66	0.150	20.04	0.132

5	3-Methyl-hexane	Alkane	CID: 11507	Y	5.43E-03	12.41	0.144	12.57	0.447	11.47	0.339	11.50	0.102	11.13	0.509
9	1-Ethyl-1-methyl-cyclopentane	Alkane	CID: 28030	Y	3.73E-04	7.54	1.018	7.75	1.752	0.00	0.000	4.03	1.281	3.93	1.023
12	Methyl-cyclohexane	Alkane	CID: 7962	Y	1.79E-01	11.96	0.411	12.42	0.625	10.04	0.775	12.00	0.431	11.53	0.739
13	Hexane	Alkane	CID: 8058	Y	1.78E-07	14.68	0.223	13.30	0.092	13.09	0.212	14.29	0.134	10.56	0.226
27	2,3-Dimethyl-hexane	Alkane	CID: 11447	Y	1.27E-07	13.00	0.306	11.21	0.054	12.38	0.118	14.88	0.082	11.84	0.169
		TOTAL ALKANES		NA	5.01E-05	59.59	1.480	57.26	2.484	46.97	1.117	56.69	1.699	49.00	1.392

14	4,5-Dimethyl-1-hexene	Alkene	CID: 27683	Y	1.04E-06	14.14	0.040	13.67	0.185	12.26	0.140	7.69	0.965	13.60	0.316
38	1-Decene	Alkene	CID: 13381	N	1.08E-02	11.03	0.072	10.95	0.029	10.78	0.356	11.38	0.064	11.05	0.040
53	7-Tetradecene	Alkene	CID: 25209	Y	1.11E-02	11.41	0.162	11.40	0.077	11.55	0.278	10.45	0.212	11.39	0.223
57	3-Dodecene	Alkene	CID: 137285	Y	5.38E-03	11.62	0.051	11.61	0.032	11.58	0.399	11.97	0.037	11.71	0.039
		TOTAL ALKENES		NA	1.01E-04	48.20	0.201	47.63	0.228	46.16	0.997	41.49	0.976	47.75	0.382

54	1-Dodecyne	Alkyne	CID: 69821	Y	1.04E-07	11.25	0.066	9.62	0.058	11.56	0.079	3.09	1.278	9.59	0.172
56	1-Undecyne	Alkyne	CID: 75249	Y	2.48E-07	11.88	0.055	12.29	0.026	10.91	0.155	10.63	0.096	10.99	0.042
		TOTAL ALKYNES		NA	4.92E-08	23.13	0.113	21.91	0.067	22.46	0.229	13.72	1.313	20.58	0.175

2	4-(acetyloxy)-2-Butanone	Ketone	CID: 139100	Y	6.70E-07	15.09	0.139	15.45	0.157	13.70	0.559	10.86	0.328	9.99	0.191
3	3-Methylisovaline	Methyl amino acid	CID: 229525	Y	1.64E-06	14.43	0.497	13.51	0.637	10.21	0.952	7.63	0.173	8.33	0.162
30	Oxime-, methoxy-phenyl	Oxime	CID: 9602988	Y	2.48E-03	12.39	0.234	11.76	0.451	10.04	0.465	11.99	0.398	10.64	0.427
40	Methyl-methoxy-hydroxymethyl-amine	Alcohol/amine	CID: 554060	Y	1.47E-08	12.59	0.046	11.91	0.024	10.56	0.154	13.50	0.072	14.92	0.058
43	2-Propenoic acid, 3-phenyl-, 2-methyl-2-propenyl	Carboxylic acid	CID: 5363458	Y	4.89E-08	13.37	0.039	12.96	0.029	12.98	0.065	12.29	0.081	10.51	0.091
44	1,2-Ethanediol, 1-phenyl	Diol	CID: 7149	Y	6.09E-08	12.11	0.084	10.72	0.069	11.79	0.074	11.02	0.091	12.89	0.141
		TOTAL OTHERS		NA	5.06E-06	79.98	0.750	76.33	1.059	69.29	1.868	67.29	0.569	67.29	0.323

1	UMUMVOC 1 (*m/z* 89)	Unknown	NA	Y	4.11E-06	15.07	0.296	15.52	0.235	11.61	1.259	7.82	0.167	7.48	0.241
4	UMUMVOC 2 (*m/z* 43, 49, 57, 61, 77, 83)	Unknown	NA	Y	1.23E-03	14.91	0.232	14.64	0.277	13.24	0.250	13.24	0.425	13.58	0.279
6	UMUMVOC 3 (*m/z* 55, 69, 89)	Unknown	NA	Y	2.02E-04	12.64	0.962	10.45	1.002	7.33	0.993	6.77	0.385	6.38	0.237
8	UMUMVOC 4 (*m/z* 49, 84)	Unknown	NA	Y	1.84E-03	13.81	0.314	13.47	0.338	11.75	0.248	12.32	0.418	12.41	0.311
17	UMUMVOC 5 (*m/z* 41, 55, 70, 91)	Unknown	NA	Y	2.12E-04	10.32	0.493	10.39	0.548	8.80	0.241	8.40	0.192	11.72	0.724
21	UMUMVOC 6 (*m/z* 41, 57, 88, 103, 133, 151, 179, 209, 229, 281)	Unknown	NA	N	8.80E-04	11.63	0.072	11.74	0.055	11.76	0.039	11.34	0.088	11.29	0.205
25	UMUMVOC 7 (*m/z* 43, 55, 70, 83, 91, 104, 151, 211, 229, 281)	Unknown	NA	Y	3.55E-03	11.01	0.160	10.95	0.136	11.08	0.084	10.60	0.121	9.86	0.293
26	UMUMVOC 8 (*m/z* 43, 55, 70, 83, 91, 104, 151, 211, 229, 281)	Unknown	NA	N	4.25E-04	12.54	0.187	12.08	0.226	11.00	0.178	11.27	0.254	11.26	0.213
31	UMUMVOC 9 (*m/z* 43, 55, 70, 84, 104, 114, 133, 151)	Unknown	NA	Y	4.57E-07	11.51	0.124	10.85	0.020	10.97	0.120	12.80	0.075	10.60	0.072
34	UMUMVOC 10 (*m/z* 41, 57, 75, 85, 103, 117, 133, 151, 209)	Unknown	NA	N	6.89E-07	10.91	0.116	10.66	0.059	10.18	0.073	11.47	0.069	10.03	0.144
49	UMUMVOC 11 (*m/z* 43, 61, 73, 88, 101, 148, 267)	Unknown	NA	Y	4.02E-08	12.60	0.079	11.23	0.031	9.84	0.203	13.48	0.090	12.53	0.116
		TOTAL UNKNOWNS		NA	1.96E-06	136.94	1.879	131.99	1.598	117.57	2.722	119.52	1.363	117.15	1.202
		TOTAL VOC		NA	2.42E-07	686.37	6.078	636.64	4.590	602.18	6.300	632.14	4.201	598.38	3.188

**Fig. 3:**
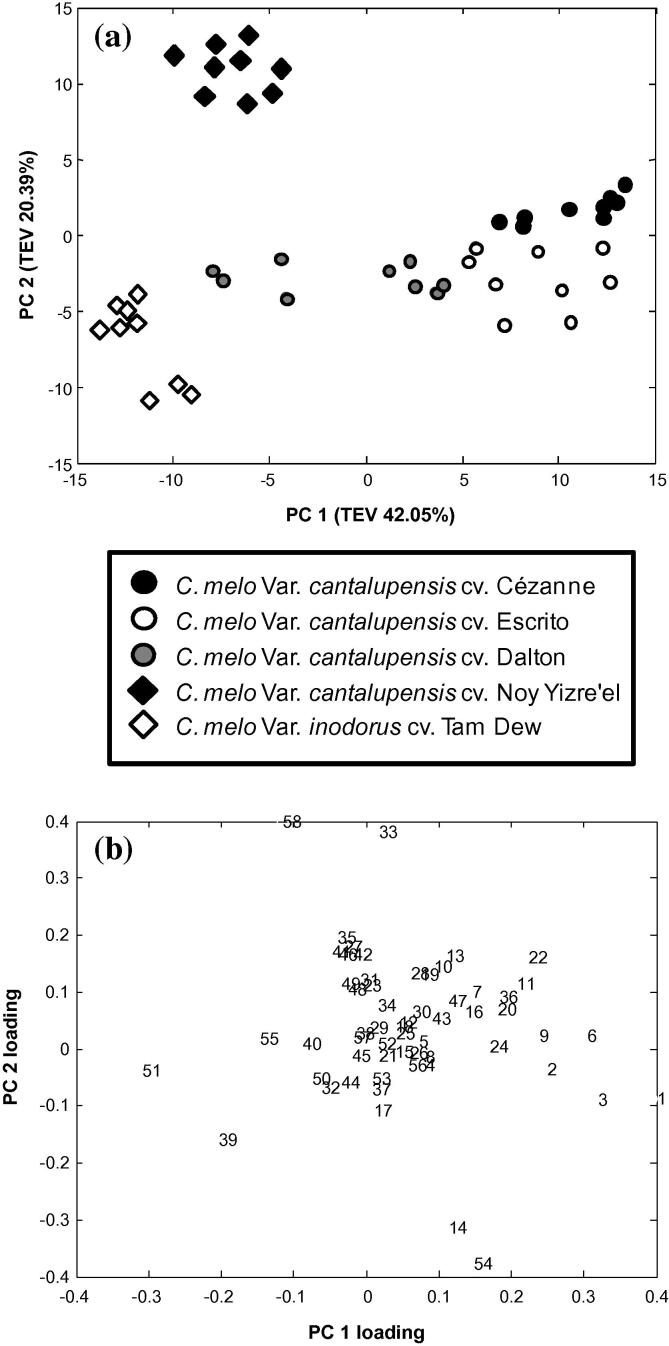
Principal component analysis of PDMS TD-GC–MS data of all melon cultivars. A PCA model was produced for the PDMS TD-GC–MS VOC data where all five melon cultivars were included, each group was based upon *n *= 9 (3 biological replicates × 3 technical replicates). The PCA model was based upon the first 5 PCs. PC1 (42.1% TEV) was plotted against PC2 (20.4% TEV) to produce a PC scores plot (a) and a PC loadings plot (b). The identifications of each of the PC reference numbers applied in the PC loadings plot (b) are given in Table 1.

Within this study a greater number of VOCs were detected by TD-GC–MS than in a previous study applying GC–FID (Flame Induced Dissociation) to the same Charentais melon cultivars (Aubert and Bourger, 2004). It is difficult to conclude whether this was associated with differences in sensitivity/selectivity between PDMS trapping and dichloromethane extraction, the sensitivity of the different detection methodologies, possible differences in the melon fruit due to growing practices, location and year, or a combination of these factors. In contrast to earlier studies that have measured the VOC content of a diverse range of *C. melo cantalupensis* varieties via typically several different extraction methodologies (e.g. Yabumoto et al., 1977, Bauchot et al., 1998), the numbers of VOCs detected here using TD-GC–MS is relatively high, whilst the chemistries of the trapped VOCs are also diverse. This VOC diversity is already sufficient for PCA to discriminate between the cultivars studied (Fig. 3). It can be concluded that the PDMS sorbent is capable of sampling a diverse range of VOCs that are suitable for the assessment of aroma qualities in fruit related foodstuffs. The newly developed method represents an alternative simplified means of accurate and robust high-throughput VOC sampling from very limited volumes of material.

### Multivariate and univariate analyses suggest substantial compositional VOC differences between the melon cultivars

Initially a multivariate statistical method, PCA (Jolliffe, 1986), was applied to the PDMS dataset in order to summarise graphically the abundance changes in VOCs between the different melon cultivars and to also assess the technical, analytical, and biological reproducibility. To prevent the VOC peaks of greatest intensity from dominating the PCA model, the data were first log_10_ transformed thus giving all VOCs a normal distribution. The PCA scores plot indicated that the levels of technical, analytical, and biological reproducibility were extremely high as assessed by the close superimposition of technical and biological replicates (Fig. 3a). The PCA scores plot (PC1 *x* PC2) clearly revealed the closer relativity of the three French Charentais melons (Cézanne, Escrito, and Dalton) when compared to the Israeli melons. The mid shelf-life Charentais, Escrito, and the short shelf-life Charentais, Cézanne, formed clusters which became respectively more distant from the Dalton melon cultivar along the PC1 axis, thus reflecting the increasing aroma and decreasing shelf-life of the Charentais melon cultivars. The Noy Yisre’el cultivar formed a distinct and distant cluster on the PC2 axis away from the three French Charentais cultivars and the non-aromatic Tam Dew cultivar. Interpretation of the PCA loadings plot (Fig. 3b) allowed for the mining of the VOC variables that contributed most greatly to the separation of the cultivar-based clusters observed in the PCA scores plot. As a second step, PCA models (PC1 *x* PC2) were derived individually for the Israeli (Supplementary Fig. 3) and French (Supplementary Fig. 4) melons, due to differences in growth conditions. Significant VOC differences between the melon cultivars became even clearer within these location based models. In addition to multivariate analyses performed with PCA, a univariate analysis (Kruskal–Wallis non-parametric N-Way ANOVA) was performed to test for significant VOC differences between the five cultivars. VOCs that were deemed as being discriminant within the PCA loadings plots (Fig. 3b, Supplementary Figs. 3b and 4b) and which were also significantly different at the 99.9% significance level according to Kruskal–Wallis testing, were deemed as significant VOCs warranting further investigation. Following this data mining regime, 52 out of the 58 detected VOCs were deemed as being significant.

### A wide range of esters and other VOC classes are significantly different between melon cultivars and also make significant contributions to melon aroma

**Fig. 4:**
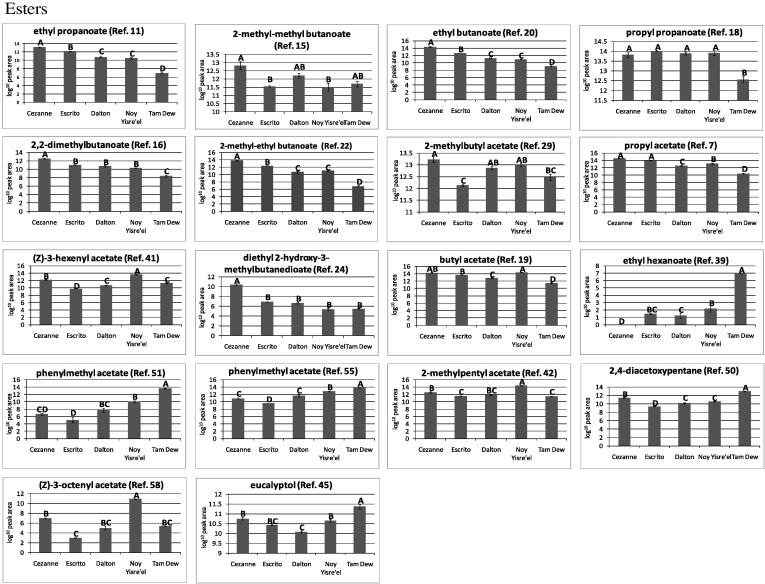

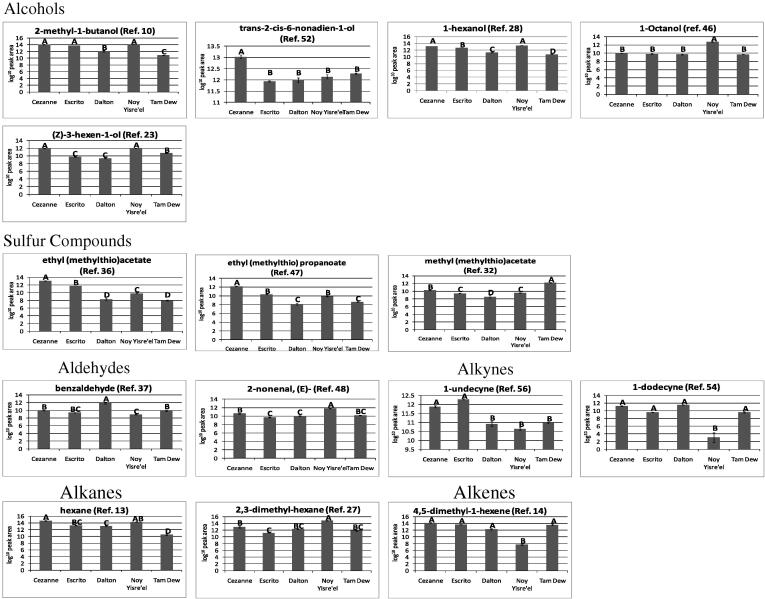
Trend plots of a selection of significant PDMS TD-GC–MS detected volatile organic compounds (VOCs). The normalised log_10_ scaled PDMS TD-GC–MS data for a selection of VOCs identified as being discriminant by PCA and Kruskal–Wallis were averaged for each melon cultivar and plotted for comparison. The error bars represent the standard error within the non-averaged data. A selection of the 31 most discriminant VOCs is presented. Letters correspond to Tukey groups: for each VOC the means with the same letter are not significantly different (*P *< 0.05).

For the assessment of the differential VOC trends highlighted by the statistical analyses, the data were first averaged by cultivar and standard errors calculated (Table 1). From the cultivar averaged data, heat maps were produced to compare the respective relatively-quantified levels of all VOCs (Supplementary Fig. 2), bar graph trend plots (Fig. 4) were also produced for the 31 most discriminative VOCs. When totalled, Cézanne had the greatest VOC content, followed by Escrito and Noy Yisre’el, Dalton and Tam Dew had the lowest VOC contents.

Esters were found to be the major class of VOCs detected in all five melon cultivars, with levels being greatest in Noy Yisre’el and Cézanne and lowest in Tam Dew and Escrito. Esters have been known to contribute highly to the aroma of ripe melons (Aubert and Bourger, 2004, Bauchot et al., 1998, Beaulieu and Grimm, 2001, Wyllie et al., 1996, Yabumoto et al., 1977) as well as other fruit such as Asian pear and pineapple (Takeoka et al., 1992, Umano et al., 1992). Within this study, the esters of greatest abundance included ethyl butanoate, propyl acetate, propyl propanoate, butylacetate, 2-methylbutyl acetate, and 2-methyl-ethyl butanoate, which despite of differences in potential trapping efficiencies of PDMS for different esters agrees with previous studies (Aubert and Bourger, 2004, Bauchot et al., 1998, Beaulieu and Grimm, 2001, Wyllie et al., 1996). Yabumoto et al. (1977) showed that the concentration of 2-methyl-ethyl butanoate increased as melon fruits ripened, this study indicates that it is of highest abundance in the most fragrant melon cultivars and is significantly reduced in the non-aromatic Tam Dew melon. In total, 13 of the 20 detected esters, were significantly reduced in the long shelf-life Dalton compared to the mid shelf-life Escrito, and were of greatest abundance in the short shelf-life Cézanne cultivar. The same 13 esters were also significantly more abundant in the Israeli aromatic Noy Yisre’el melon than the non-aromatic Tam Dew. The abundances of esters in Noy Yisre’el were typically similar to those reported in the Cézanne and Escrito Charentais melons, with the exceptions of (Z)-3-octenyl acetate, phenylmethyl acetate, 2-methylpropyl butanoate and propyl propanoate, which were all more abundant in Noy Yisre’el than any of the Charentais cultivars. The esters which revealed the most reduced abundances in the non-aromatic Tam Dew when compared to the four aromatic cultivars included ethyl propanoate, 2-methyl-ethyl butanoate, propyl propanoate, and 2-methylpropyl butanoate. Interestingly, phenylmethyl acetate (imparts an apple/pear note), ethyl hexanoate and eucalyptol, were all of much greater abundance in the non-aromatic Tam Dew than in any of the aromatic cultivars.

Within this study only three sulfur compounds were detected via trapping upon PDMS in contrast to eight detected via dichloromethane extraction of the same melon cultivars and analysis by GC–FID (Aubert and Bourger, 2004). The impact upon flavour and aroma as orchestrated by sulfur compounds has been well characterised across a number of fruit species including melon (Buttery et al., 1982, Gonda et al., 2013, Homatidou et al., 1989, Wyllie and Leach, 1992, Wyllie et al., 1994), Asian pear (Takeoka et al., 1992), pineapple (Umano et al., 1992), and strawberry (Dirinck et al., 1981). Interestingly, all three detected sulfur compounds were significantly reduced in abundance in the long shelf-life Charentais Dalton melon compared to the medium shelf-life Escrito and short shelf-life Cézanne. For the Israeli aromatic Noy Yisre’el melon, the abundances of two sulfur compounds, ethyl (methylthio) acetate and ethyl (methylthio) propanoate, were very similar to the mid shelf-life Escrito melon. The abundance of ethyl (methylthio) acetate and ethyl (methylthio) propanoate were reduced in the non-aromatic Tam Dew compared to Noy Yisre’el. Interestingly, the third sulfur compound, methyl (methylthio) acetate, was greater in abundance in the non-aromatic Tam Dew compared to the four other cultivars. Despite this observation it was also reduced in abundance from the short to long shelf-life French Charentais. Aubert and Bourger (2004) detected many sulfur compounds of much higher concentration than ethyl (methylthio) propanoate within the same melon cultivars, this may reflect that PDMS is highly selective towards specific sulfur compounds, or that the sulfur compounds varied greatly between growth practices and year in France.

A number of alcohols were also identified as being significantly different in abundance between the five melon cultivars, these included 2-methyl-1-butanol, (Z)-3-hexen-1-ol, 1-hexanol, 1-octanol, and trans-2-cis-6-nonadien-1-ol which is also known as cucumber alcohol. Trans-2-cis-6-nonadien-1-ol was much more abundant in Cézanne than any other melon cultivar, which is of interest since classically high levels of trans-2-cis-6-nonadien-1-ol have been associated with immature fruit where it is known to impart a cucumber like aroma (Beaulieu and Grimm, 2001). 1-octanol revealed similar abundances in all melon cultivars with the exception of Noy Yisre’el which showed an increase in abundance. 2-methyl-1-butanol, (Z)-3-hexen-1-ol, and 1-hexanol, were all of greatest abundance in Cézanne and Noy Yisre’el, they were significantly reduced in all three other cultivars.

A series of VOCs belonging to a diverse range of chemical classes including ketones, aldehydes, alkanes, alkenes, and alkynes, were also identified within this study. The aldehyde, 2-nonenal, was detected at quite high but yet similar abundance across all melon cultivars with the exception of Noy Yisre’el where it was greater in abundance. It is thought that the combination of aldehydes such as 2-nonenal along with high levels of an array of different esters and sulfur compounds are largely responsible for the aroma of melon fruit (Yabumoto et al., 1977). Aldehydes are known to serve as key aroma compounds in fruits where they impart a cucumber-like flavour (Kemp et al., 1972, Kemp et al., 1974), they are also known to be of higher concentration in immature melon fruit, whereas esters are more abundant in ripe melon fruit (Beaulieu and Grimm, 2001). Likewise ketones are thought to be of greatest concentration in immature fruit and decrease as the fruit ripens. The majority of compounds classed as alkanes, alkenes, alkynes, others, or unknowns, were found to be less abundant in the long shelf-life Charentais cultivars than the medium and short shelf-life cultivars, likewise they were also commonly reduced in the Israeli non-aromatic Tam Dew compared to Noy Yisre’el.

### Quantification of amino acids by ^1^H NMR suggests a contribution to melon aroma

**Fig. 5:**
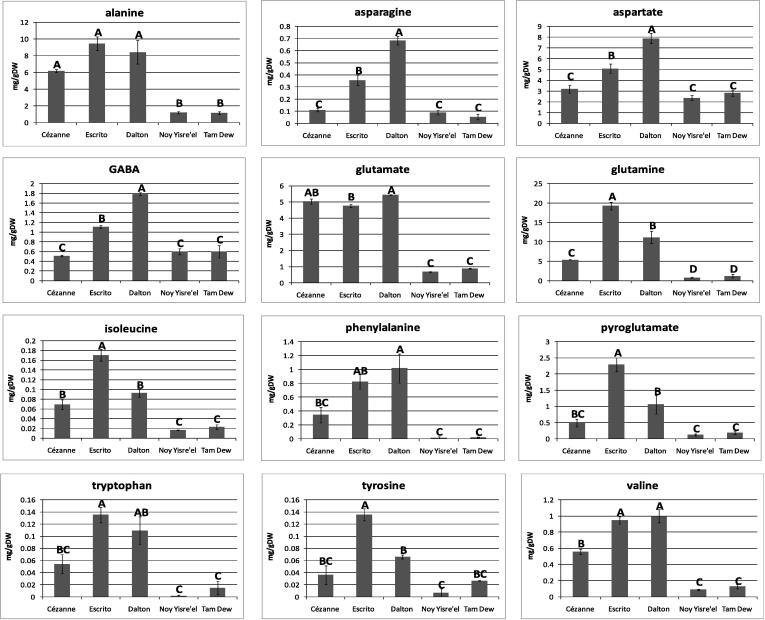
Amino acid quantification by ^1^H NMR in five melon cultivars. The quantified amino acid levels (mg/gDW), as detected by ^1^H NMR of polar extracts, were averaged for each melon cultivar and plotted for comparison. The error bars represent the standard error within the non-averaged data. Letters correspond to Tukey groups: for each amino acid the means with the same letter are not significantly different (*P *< 0.05).

The biosynthetic pathway of a great number of plant volatile constituents can be traced back to primary metabolism, with carbohydrates, fatty acids, and especially amino acids, representing the natural carbon pools for fruit aroma. A great range of alcohols, aldehydes, esters and sulfur compounds are known to be derived from the degradation of aromatic and branched chain amino acids (Gonda et al., 2013, Schwab et al., 2008). Branched chain amino acids such as isoleucine are known to be precursors for esters such as 2-methylbutyl acetate and 2-methyl-methyl butanoate, whereas aromatic amino acids such as phenylalanine through decarboxylation reactions can form alcohol and aldehyde volatile constituents (Schwab et al., 2008). With respect to this we sought to quantify the amino acid levels by ^1^H NMR and consider their potential contribution to the VOC levels detected within the various melon cultivars. Typically all 12 of the detected amino acids were 3–10-fold greater in concentration within the three French Charentais melons than in the Israeli Tam Dew and Noy Yisre’el cultivars. Previous analyses have suggested that the levels of amino acids in greenhouse grown fruit are significantly lower than in the same fruits cultivated outdoors making the direct comparison of French and Israeli fruit within this study difficult. The amino acids of greatest concentration (2–20 mg/gDW) included alanine, aspartate, glutamine, and glutamate, the amino acids of intermediate concentration (0.2–2 mg/gDW) included gamma-aminobutyric acid (GABA), valine, pyroglutamate and phenylalanine, the amino acids of lowest concentration (0.01–0.2 mg/gDW) included asparagine, isoleucine, tryptophan and tyrosine (Fig. 5). Despite amino acid levels being significantly lower in the Israeli cultivars than the French Charentais melons, it was apparent that the aromatic Noy Yisre’el melon contained lower levels for a number of specific amino acids, including aspartate, glutamate, glutamine, isoleucine, valine, tryptophan, and tyrosine, than the non-aromatic Tam Dew. Likewise, the lesser aromatic French Charentais melons, Escrito and Dalton, contained much greater concentrations of all 12 detected amino acids (with the exception of glutamate) than the highly aromatic Cézanne (Fig. 5). This reduction in amino acid content clearly correlates with increased levels of VOCs and thus increased levels of melon fragrance. By performing time course studies across the fruit development period as well as controlled isotopic flux experiments (as difficult although not impossible as this may be in melon fruit) it will be possible to investigate the contribution made by specific amino acids or classes of amino acid to the fruits ester and alcohol volatile constituents and how these volatiles alter throughout fruit development. Such an experiment could potentially provide a much greater knowledge of the fruit volatile constituents and pathways by which they are formed, as well as the development of aroma during fruit ripening.

## Experimental

### Plant materials

The French melon cv. Cézanne, Escrito and Dalton, are commercial F1 hybrids. Seeds were obtained from Clause-Tézier (FR). Plants were grown by CEFEL in an open field in the South-West of France (Moissac, 44°N × 1°E) between April and September 2007. The three melon varieties were cultivated according to commercial practices in South-West France with a specific planting date for each variety, and thus also a specific harvest period. Cézanne, a variety adapted to the early growing season in the south west of France, was planted on the 11th April 2007, Escrito, a variety adapted to the normal-to-late season, was planted on the 22nd of May 2007, and Dalton, a variety adapted to the late growth season, was planted on the 28th June 2007. The soil type was clay and limestone, the plant density was 9200 plants/ha. The Israeli melon cv. Noy Yisre’el and Tam Dew, were obtained from the germplasm collection at the Agricultural Research Organisation (ARO), Volcani Centre (IL). Plants were grown in a green house (32°N × 35°E) in volcanic tuff and peat (1:1), at a plant density comparable to 20,000 plants/ha. Seedlings were planted on the 15th June 2006 and ripe fruit were harvested at about 35 days after pollination. For all cultivars, cultivation, irrigation, watering, fertilisation and pathogen-pest control were performed according to the respective local commercial practices.

Melon fruit were harvested at commercial maturity of each variety based upon the peel color and aspect of the pedicel abscission zone. The experimental design for cultivation and harvest was directed by standard cultivation practice to ensure optimal conditions for each individual variety in relation to delivering consumer-relevant materials. Therefore the date of harvest of the various melon varieties differed according to their growth season and date of planting. Care was taken to harvest fruits from each variety at comparable physiological stages of maturation. Biological variation was compensated for by collecting multiple fruits for each variety when these were at the optimum moment for harvest, always in the morning of each harvest day.

The three melon varieties cultivated in the south west of France, Cézanne, Escrito, and Dalton, were harvested upon July 5th, August 9th, 17th September 2007, respectively, resulting in growth periods of 85, 79, and 81 days, respectively. The two Israeli melon varieties, Noy Yisre’el and Tam Dew, were harvested upon the same day, 8th September 2006, resulting in a growth period of 85 days. Fruits were therefore always harvested at a physiologically comparable stage of fruit maturation, as verified by experts of CEFEL-France, and ARO-Israel. Melons were transported in insulated boxes and upon arrival, processed within 2 h. Fruits were selected depending on the size, weight and colour in order to make three homogeneous lots (biological replicates), each made up of pools of six fruit. The fruits were first washed for 1–2 min with tap water and air dried. Each melon was quartered, two opposing quarters were selected, the skin and hard outer flesh were removed. The remaining flesh was cut in 2 × 2 cm cubes and immediately frozen in liquid nitrogen and stored at −80 °C until grinding. All samples were ground (French melons; UMC5 grinder, STEPHAN™, Lognes, FR: Israel melons; IKA A11 grinder, IKA Werke, Staufen, DE) in liquid nitrogen to a homogeneous fine frozen powder and stored at −80 °C. The ground samples were shipped on dry ice to the different analytical laboratories and stored at −80 °C on receipt. Sample extraction and analysis was undertaken within six months of sample receipt.

### Preparation of polydimethylsiloxane (PDMS) patches for VOC sampling

PDMS patches (20 × 15 × 50 mm) were cut from a single sheet (Goodfellow Cambridge Ltd, Huntingdon, UK). The PDMS patches were conditioned as described in Xu et al. (2010), transferred directly into clean thermal desorption (TD) tubes (Markes international Ltd, Llantrisant, UK) and capped. The TD tubes were stored at room temperature within an airtight glass container filled with a layer of activated molecular sieves (Sigma Aldrich Ltd, Dorset, UK). The TD tubes were used for sampling and analysis within 48 h of conditioning. A random selection of the TD tubes (1:5) were analysed to confirm that the batch was clean and free from contaminants before use.

### Sampling melon VOCs with PDMS patches

For each melon sample, 200 μL of frozen melon homogenate was transferred into a 10 mL screw top sampling vial (Chromacol Ltd, Hertfordshire, UK). Three PDMS patches were removed from their TD tubes and suspended from a solvent cleaned nickel plated ‘treble fishing hook’. The fishing hook was attached to the silicon insert within the screw top cap. This sampling setup (Fig. 1) permitted the collection of three technical replicate samples per biological sample. To the 200 μL melon homogenate, 1 μL of 0.0001% (v/v) 1-pentanol (Sigma–Aldrich Ltd, UK) diluted in dichloromethane (DCM) was added. The 1-pentanol was utilised as an internal standard to assess the recovery of VOCs on each PDMS patch, thus allowing for the removal of errors introduced by the passive VOC sampling method. Once capped, the sampling vials were placed into a dry block heater fitted with appropriate blocks for 10 mL vials. The dry block heater had been heated to 40 °C in advance. Each sample vial was subjected to heating for 30 min. Once the sampling was complete, the patches were quickly placed back into TD tubes. All samples were analysed immediately after sampling took place. Sampling and analysis for all five melon cultivars was performed in a randomised order.

### Thermal desorption-gas chromatography–mass spectrometry (TD-GC–MS) analysis of VOCs

A Markes Unity 1 TD unit (Markes International Ltd, UK) was connected directly through the front injector assembly of a Varian CP 3800 gas chromatograph-2200 quadrupole ion trap mass analyser (Varian Inc, Oxford, UK). The unity transfer line was connected directly to the analytical column within the GC oven through the use of a Valco zero dead volume micro union (Thames-Restek Ltd, Buckinghamshire, UK). The cold trap packing material was Tenax-TA carbograph 1 TD (Markes International Ltd, UK). The transferline to the GC was maintained at 150 °C isothermally. The analytical column employed was an Agilent HP-5 60 m × 0.25 mm (I.D.) with a film thickness of 0.25 μm (Agilent Technologies Ltd, Berkshire, UK). To ensure spiltless injection and efficient desorption within the Markes Unity 1 TD unit cold trap, a minimum flow rate of 1.5 mL/min of helium gas was required. The on column pressure was adjusted to 85.5 kPa.

The thermal desorption profile involved sample desorption at 180 °C for 3 min whilst maintaining the cold trap at −10 °C prior to heating to 300 °C for 3 min. The GC method employed helium as the carrier gas at a flow rate of 1 mL/min, initially the temperature was set to 50 °C for a hold time of 7.5 min and then increased to 230 °C at a ramp rate of 4 °C/min with no final hold. The transferline to the MS was maintained at 270 °C isothermally. The MS was maintained at 200 °C, an electron impact ionisation source was utilised at 70 eV, the MS was set to scan from *m/z* 40–400 at a scan rate of 1.03 scans/s. Cold trap blanks and column blanks were run after each sample to ensure that the system was free of artefacts before analysis of the next sample commenced.

### Solid phase micro extraction-GC–MS analyses of melon VOCs

For comparison of the newly devised TD-GC–MS analytical method, the two Israeli and two of the three French cultivars were also subjected to an established SPME-GC–MS method. The extraction and analytical procedures used, as well as the data processing workflow within the MetAlign software package, were as described previously by Moing et al. (2011) and Verhoeven et al. (2011). The netCDF files have been deposited, with associated metadata, into the Metabolomics Repository of Bordeaux MeRy-B (http://services.cbib.u-bordeaux2.fr/MERYB/public/PublicREF.php?REF=M08004).

### TD-GC–MS data processing, deconvolution and identification of VOCs

All TD-GC–MS data were recorded as Varian sms files, the files were converted to netCDF using the Palisade MASSTransit program (Scientific Instrument Services Inc, NJ, USA). The netCDF files have been deposited, with associated metadata, into the Metabolomics Repository of Bordeaux MeRy-B (http://services.cbib.u-bordeaux2.fr/MERYB/public/PublicREF.php?REF=M08004). The netCDF files were then loaded into Matlab R2006 (The MathWorks Inc, Natick, MA, USA) where the mexnc toolbox was utilised for data processing. Alignment of the data was performed by correlation optimised warping (COW) (Nielsen et al., 1998). The two parameters of COW, number of segments and slacking size, were optimised by using a simplex optimisation procedure as described by Skov et al., 2006. A detailed description of the COW based alignment and deconvolution procedure is presented in Xu et al. (2010). The signal derived from the 0.0001% 1-pentanol internal standard (based upon the peak area in single ion monitoring mode) was stable throughout the experiment for all technical and biological replicate samples regardless of differences between sample matrices or cultivars (Fig. 2). For this reason, it was not necessary to normalise to the internal standards response. The deconvoluted mass spectra were exported as NIST compliant text files into the NIST MS search 2.0 software, putative compound identification was based upon qualitative similarity matching against the NIST 02 mass spectral library. An overall match score of greater than 70% based upon the combined average from forward and backward library searches was used as an initial match criteria. Mass Spectral matches were then checked by visual interpretation thus giving high confidence for each of the VOC identifications (MSI Level 2: Sumner et al., 2007). The resulting deconvoluted peak table was next subjected to chemometric analyses.

### Chemometric analyses of melon TD-GC–MS VOC data

Within MatLab R2006, classical PCA was performed according to the NIPALS algorithm (Jolliffe, 1986) as previously described in Allwood et al. (2006) and Biais et al. (2009). Scores plots and loadings plots were generated for the principal components (PCs), as were text files that were exported for all of the PC loadings. The results of PCA were visually interpreted. In addition to PCA, to aid with biological interpretation of the TD-GC–MS data, a heat map was generated for the VOC data averaged for each melon cultivar using Multi Experiment Viewer 4.41 (Saeed et al., 2003), a heat map was also generated following the same method for the SPME-GC–MS data. A non-parametric univariate significance test, the Kruskal–Wallis test, was also performed within MatLab R2006 as described within the MatLab handbook. Variables that were significant at a 99.9% confidence limit and which passed a 5% False Discovery Rate (FDR) were further investigated. A Tukeys test was also performed at the 95% confidence limit within XLSTAT software (Addinsoft, Paris, FR).

### Extraction and ^1^H NMR analysis for amino-acid quantification

The polar metabolites were extracted from the frozen ground melon samples, and extracts were titrated and lyophilised, as described by Biais et al. (2009) and Moing et al. (2011). To the lyophilised titrated extract 500 μL of D_2_O with sodium trimethylsilyl [2,2,3,3-^2^H_4_] propionate (TSP, 0.01% final concentration for chemical shift calibration) were added and centrifuged at 10,000*g* for 5 min and the supernatant was transferred to a 5 mm NMR tube. ^1^H NMR spectra were acquired and the raw data processed as described previously (Biais et al., 2009, Moing et al., 2011). Resonance identification was performed by comparison to published data (Fan, 1996, Moing et al., 2004), spectra of plant extracts (http://www.cbib.ubordeaux2.fr/MERYB) and spectra of reference compounds acquired under the same conditions (local database), and by standard spiking. For absolute quantification three calibration curves (glucose: 2.5 to 100 mM, glutamate and glutamine: 0 to 30 mM) were prepared and analysed under the same conditions. The glucose calibration was used for the quantification of all amino-acids other than glutamate and glutamine that were quantified using their own calibration curve. The metabolite concentrations were calculated using AMIX (version 3.7.3, Bruker, Karlsruhe, DE) and Excel (Microsoft, WA, USA) software. A Tukey’s test was performed at the 95% confidence limit within XLSTAT software (Addinsoft, FR). The raw ^1^H NMR spectral profiles have been deposited, with associated metadata, into the Metabolomics Repository of Bordeaux MeRy-B (http://services.cbib.u-bordeaux2.fr/MERYB/public/PublicREF.php?REF=M08004).

## Concluding remarks

This study has clearly indicated the validity of a novel and high-throughput ambient VOC sampling methodology that consists of a simple arrangement of sampling equipment and which is easy to perform and highly reproducible for very limited volumes of sample material. The method appears to be suitable for the high-throughput screening of fruits and other plant derived food stuffs based upon aromatic quality related compounds such as esters, aldehydes, sulfur compounds and alcohols. Despite PDMS being biased towards the trapping of polar VOCs, a diverse range of VOC chemistries and a large number of compounds were trapped and detected. The results clearly demonstrate that volatile compounds discriminate between the French long shelf life, mid shelf life, and “wild” short shelf life Charentais cultivars. The results also illustrate that the Israeli aromatic Noy Yisre’el melon has a similar VOC profile to the mid shelf life French Charentais melons, whereas the non-aromatic Tam Dew largely has significantly reduced VOC components compared to the other four melon cultivars. A large number of the significant VOCs from melon detected within this study have previously been shown to be key components within the aroma of melon and other fruits. The data presented within this study justifies further investigations of melon fruit and how the aroma profile changes during fruit development and ripening, as well as the analysis of VOCs it will also be critical to measure amino acid content and ethylene levels, as well as performing flux based analyses, to elucidate more fully how aroma associated volatiles are formed and which amino acids serve as their pre-cursors across the various fruit developmental stages.

## Financial Support

J.W.A. and all authors would like to thank the EU for experimental funding within the META-PHOR project (FOOD-CT-2006-036220). R.G. would like to thank the UK BBSRC and EPSRC (BBC0082191) for financial support of the MCISB (Manchester Centre for Integrative Systems Biology). R.D.H and R.C.H.D.V. acknowledge the Centre for Biosystems Genomics, which is part of the Netherlands Genomics Initiative, for additional funding. A.M., C.D. and M.M. would like to thank the Metabolome Facility of Bordeaux Functional Genomics Centre for support.
