# Supplementary material for: Metabolomics in melon: A new opportunity for aroma analysis
Source: Phytochemistry. 2014 Mar;99:61–72. doi: 10.1016/j.phytochem.2013.12.010 (PMC4180013; doi:10.1016/j.phytochem.2013.12.010)
Supplement: Supplementary Figs. 1–4 [file mmc1.docx]

**Supplementary Figures**

**Supplementary Figure 1: Heat map of normalised log_10_ scaled SPME-GC-MS data to illustrate volatile organic compound (VOC) differences between four melon cultivars**

A heat map was produced for 101 of the SPME-GC-MS detected VOC features based upon the log_10_ scaled data to allow easy comparison of their relatively-quantified levels between melon cultivars.

**Supplementary Figure 2: Heat map of normalised log_10_ scaled PDMS TD-GC-MS data to illustrate volatile organic compound (VOC) differences between all melon cultivars**

A heat map was produced for all the PDMS TD-GC-MS detected VOC features based upon the log_10_ scaled data to allow easy comparison of their qualitative levels between the five melon cultivars.

**Supplementary Figure 3: Principal component analysis of PDMS TD-GC-MS data to compare the aromatic Noy Yisre’el cultivar with the non-aromatic Tam Dew melon cultivar**

A PCA model was produced based upon PDMS TD-GC-MS detected VOCs where the aromatic Noy Yisre’el cultivar was compared with the non-aromatic Tam Dew cultivar, each group was based upon *n*=9 (3 biological replicates x 3 technical replicates). The PCA model was based upon the first 5 PCs. PC1 (60.8% TEV) was plotted against PC2 (17.2% TEV) to produce a PC scores plot (a) and a PC loadings plot (b). The identifications of each of the reference numbers applied in the PC loadings plot (b) are given in Table 1.

**Supplementary Figure 4: Principal component analysis of PDMS TD-GC-MS data of the three French Charentais melon cultivars**

A PCA model was produced based upon PDMS TD-GC-MS detected VOCs where only the three French melon cultivars were included, each group was based upon *n*=9 (3 biological replicates x 3 technical replicates). The PCA model was based upon the first 5 PCs. PC1 (51.3% TEV) was plotted against PC2 (15.9% TEV) to produce a PC scores plot (a) and a PC loadings plot (b). The identifications of each of the reference numbers applied in the PC loadings plot (b) are given in Table 1.

**Supplementary Figure 1: Heat map of normalised log_10_ scaled SPME-GC-MS data to illustrate volatile organic compound (VOC) differences between four melon cultivars**

**Supplementary Figure 2: Heat map of normalised log_10_ scaled PDMS TD-GC-MS data to illustrate volatile organic compound (VOC) differences between all melon cultivars**

**Supplementary Figure 3: Principle component analysis of PDMS TD-GC-MS data to compare the aromatic Noy Yisre’el cultivar with the non-aromatic Tam Dew cultivar**

**Supplementary Figure 4: Principle component analysis of PDMS TD-GC-MS data of the three French Charentais melon cultivars**
